# Immunoassay standardization for the detection of immunoglobulin A (IgA) against *Porphyromonas gingivalis* antigens in saliva of individuals with and without leprosy

**DOI:** 10.1186/s13568-021-01312-7

**Published:** 2021-11-18

**Authors:** Mariana Costa Calheira, Soraya Castro Trindade, Michelle Miranda Lopes Falcão, Luciana Sales Conceição Barbosa, Gislene Regina Batista Carvalho, Paulo Roberto Lima Machado, Isaac Suzart Gomes-Filho, Elisangela de Jesus Campos, Paulo Cirino de Carvalho-Filho, Márcia Tosta Xavier, Antonio Pedro Fróes de Farias, José Tadeu Raynal Rocha Filho, Johelle de Santana Passos-Soares

**Affiliations:** 1 Immunology Department, Federal University of Bahia, Salvador, Brazil; 2 Preventive Dentistry Department, Federal University of Bahia, Salvador, Brazil; 3 Health Department, Feira de Santana State University, Feira de Santana, Brazil; 4 Immunology Service, Professor Edgar Santos University Hospital, Federal University of Bahia, Salvador, Brazil; 5 Oral Biochemistry Laboratory, Health Sciences Institute, Federal University of Bahia, Salvador, Brazil; 6 Dental School, Bahiana School of Medicine and Public Health, Salvador, Brazil; 7 Immunology and Molecular Biology Laboratory, Federal University of Bahia, Salvador, Brazil

**Keywords:** Leprosy, Periodontitis, Saliva, Immunoassay protocols, Enzyme-linked immunosorbent assay, *P. gingivalis*

## Abstract

Leprosy reactions are immune processes that cause neural damage in individuals with leprosy. As periodontitis is an infectious disease related to its development, specific antibodies to periodontal pathogens must be evaluated to better understand the humoral mechanisms underlying this relationship. Therefore, the objective of this study was to standardize an immunoassay to measure IgA specific to *P. gingivalis* antigens in the saliva of individuals with leprosy. An ELISA checkerboard titration was performed. A validation test involving 53 individuals with leprosy, 24 with and 19 without periodontitis, was conducted and a ROC curve constructed to calculate sensitivity and specificity. The coefficient of the optical densities was 2.21 and 2.66 for *P. gingivalis* crude extract and the recombinant protein HmuY, respectively. Sensitivity and specificity for the *P. gingivalis* crude extract were 66.7% and 73.7%, respectively, and for HmuY, were 62.5% and 52.6%, respectively. Specific recognition of *P. gingivalis* occurred predominantly in individuals with periodontitis, which validates the use of this test for studying periodontitis in individuals with leprosy.

*Trial registration* CAEE 64476117.3.0000.0049, 21/07/2017, retrospectively registered

## Key points

- Standardize immunoassay to measure IgA specific for *P. gingivalis* antigens.
- ELISA-HmuY showed satisfactory levels of sensitivity and specificity.
- Promising for epidemiological research in individuals with periodontitis.

## Introduction

Leprosy is a chronic infectious disease caused by *Mycobacterium leprae* which affects the peripheral nervous system of the skin. It occurs mainly in intertropical zones, such as India, Brazil, Indonesia, Bangladesh and Ethiopia, these countries being responsible for 80% of all worldwide cases (Schreuder et al. 2016; Odriozola et al. 2017). Compliocations resulting from this disease, called leprosy reactions, can cause incapacities if not treated early (Naaz et al. 2017; Odriozola et al. 2017).

The most common form of transmission occurs as a result of direct and prolonged physical contact with a diseased individual, through inhalation of *Mycobacterium leprae*, or leprosy bacillus, via the airways (Brasil 2016). Leprosy is a spectral disease classified according to the type and degree of the host’s specific cellular immunity to the bacillus. There is a clinical variability among individuals with leprosy, ranging from the tuberculoide form to the lepromatose form of the disease (Gaschignard et al. 2016; Odriozola et al. 2017). The tuberculoide form is characterized by a small number of cutaneous anesthetic lesions with well-defined and elevated borders and negative bacilloscopy. It also presents early damage to the peripheral nerve and a Th1 cell-mediated immune response. In contrast, the lepromatose form is characterized by numerous infiltrated skin lesions, high bacillary levels, damage to the peripheral nerve and a Th2 cell-mediated immune response (Walker and Lockwood 2006; Gaschignard et al. 2016).

As leprosy progresses, or even after its treatment, individuals can suffer leprosy reactions, present in approximately 10–50% of cases, especially in its multibacillary forms (Teixeira et al. 2010). Leprosy reactions are acute inflammatory manifestations that are influenced by factors such as stress, pregnancy and concomitant coinfections (Teixeira et al. 2010; Motta et al. 2011). Odontological infections, such as periodontitis, have been mentioned as potential triggers (Almeida et al. 2013).

Periodontitis is an inflammatory disease initiated by the presence of dental biofilm, involving a dysregulation of the immune response (Papapanou et al. 2018). This oral disease is characterized by the destruction of the tissues that support the teeth and is modulated by the individual’s immunoinflammatory response to perio-pathogens and their products (Garlet 2010; Śmiga et al. 2015). Its presence can exacerbate the systemic condition as a result of the liberation and increase in the concentration of proinflammatory mediators and immune complexes at a systemic level (Cardoso et al. 2018). *Porphyromonas gingivalis* have been considered as a keystone pathogen (Chapple et al. 2017; Mira et al. 2017), associated with worsening clinical periodontal measurements, such as deepening periodontal bags, radiographic measurements and alveolar bone loss (Mysak et al. 2014). This bacterium possesses a diversity of virulence factors, such as HmuY, a membrane-associated protein important to the uptake of heme from the microenvironment. These antigens can promote immunogenicity in the host tissues by stimulating innate and adaptive immunity, including the humoral response (Olczak et al. 2015).

The quantification of the concentration of periodontal markers in saliva has been one of the forms commonly used to track diseases and vulnerability in population groups (Bachrach et al. 2008). Some studies have shown greater concentrations of antibodies and inflammatory mediators, salivary immunoglobin A (IgA) for example, in individuals with periodontitis when compared to those without periodontitis, or undergoing treatment for the disease (Pudakalkatti and Baheti 2015; Gadekar et al. 2018). Thus, it can be suggested that these antibodies may be potential tracking, diagnostic and control markers for this periodontal disease. Therefore, analysis of IgA specific to *P. gingivalis* in individuals with leprosy will allow the understanding the humoral processes involved in the relationship between periodontitis and leprosy reaction. It is important to point out that saliva-based tests are simple to collect as they are non-invasive (Bachrach et al. 2008).

In this sense, the detection of specific antibodies specific to *P. gingivalis* antigens in saliva can assist in the investigation of the relationship between periodontitis and leprosy reactions in individuals with leprosy. It is important to note that there is insufficient evidence to sustain the interaction mechanism between these periodontal pathogen induced markers and leprosy reactions. As such, it continues to be a hypothesis that requires further study (Motta et al. 2011).

Thus, the present study puts forward an enzymatic immunoassay (ELISA) to evaluate the presence of periodontitis, through a humoral response mediated by IgA antibodies specific for *Porphyromonas gingivalis* antigens in the saliva of individuals with leprosy. It is expected that the specificity of salivary IgA for Porphyromonas gingivalis antigens in individuals diagnosed with leprosy will be greater in those with periodontitis.

## Materials and methods

### Study design

The ELISA was standardized in two stages. The first was a checkerboard titration with supernatant pools containing the saliva of 10 individuals with leprosy (L^+^), classified according to the presence (P^+^) or absence (P^−^) of periodontitis. Two supernatant pools containing the saliva of 6 individuals without leprosy (L^−^) were also prepared at this stage, and subsequently classified according to the presence (P^+^) or absence (P^−^) of periodontitis, so as to provide a titration parameter for individuals without leprosy, and in the same conditions as the individuals with the disease. The second stage consisted of a validation test with 53 individuals with leprosy, 24 with periodontitis and 19 without.

### Selection of participants

The participants with a leprosy diagnosis were recruited at the Dermatology Service of Professor Edgar Santos University Hospital (HUPES), in Salvador, Bahia, Brazil. Those without leprosy diagnosis were recruited at the Odontology School of the State University of Feira de Santana, Feira de Santana-Bahia, Brazil. This study was approved by the HUPES research and ethics committee (CAEE 64476117.3.0000.0049).

The individuals invited to participate in the study had a minimum age of 18 years, with no history of pregnancy, smoking habit, neoplasms, HIV-AIDS, and who were able to understand and answer the questionnaires. Also, the participants could not have undergone periodontal treatment in the 6 months prior to the study. All individuals with leprosy were recieving or had completed treatment with polychemotherapy (PCT). The individuals without leprosy had not used antibiotics and/or anti inflammatories in the last 6 and 3 months, respectively, before the periodontal exam.

The leprosy diagnosis was made by a team of dermatologists from HUPES, based on anamnesis and a dermatoneurological exam that evaluated the presence of thickened peripheral nerves and/or skin lesions or areas of the skin that were painful and/or with altered thermal and/or tactile sensitivity (Brasil 2016; WHO 2017). All cases were confirmed through bacilloscopy and an anatomopathological exam, and the individuals classified: leprosy group (L^+^) and non-leprosy group (L^−^) (WHO 2017).

The periodontal exam was performed by a trained dentist and included pocket depth probing, clinical attachment level and bleeding on probing. A Kappa test was used to evaluate intraexaminer concordance, calculated using the measures for recession and probing depth (difference of ± 1 mm) of 10% of the sample, which were 0.81 and 0.84, respectively.

Individuals who had probing depth less than 4 mm and clinical attachment less than or equal to 1 mm for all teeth, and less than 25% of bleeding on probing, were considered as not having periodontitis (Gomes-Filho et al. 2018). Individuals with severe periodontitis were selected to compose the periodontitis group of the checkerboard test, which included those with at least 4 teeth with at least one site that showed a clinical attachment level of (CIL) ≥ 5 mm, probing depth (PD) ≥ 5 mm and bleeding on probing (in the same tooth).

Individuals with moderate periodontitis were included for the validation test, composed of those who presented at least 4 teeth with at least one site with CIL ≥ 3 mm, PD ≥ 3 mm and bleeding upon probing (in the same tooth). Thus, individuals with moderate and severe periodontitis composed the group with periodontitis (P^+^), while individuals clinically healthy composed the group without periodontitis (P^−^).

### Saliva collection

Saliva collection was carried out after a confirmed 2-h fasting period. First, the individual chewed a parafilm for one minute and the saliva produced discarded. Following this, the individual chewed for another 5 min and the saliva produced collected at one minute intervals in a beaker using a funnel to help (Krasse 1988). The saliva collected was stored in a microcentrifuge tube with 1 µL of 0.1 M phenyl methane sulfonyl fluoride (PMSF) protease inhibitor (SIGMA-ALDRICH, Saint Louis, USA) (Bachtiar et al. 2020).

### Obtaining *Porphyromonas gingivalis* antigens

The immunogenic extract of *Porphyromonas gingivalis*, strain ATCC 33277 (Taxonomy NCBI ID: 431947) was produced according to the standard protocol established by Trindade et al. (2012).

The recombinant protein HmuY was produced, purified and characterized from *Escherichia coli*, using a plasmid as the cloning vector in accordance with the protocol standardized by Olczak et al. (2006).

### ELISA checkerboard

For analysis, the saliva was unfrozen and centrifuged at 2000×*g* for 10 min (Franca et al. 2007). Next, these supernatants were grouped according to the diagnosis of leprosy and periodontitis. In this way, two pools were obtained, one containing the samples of 05 individuals with leprosy and without periodontitis (L^+^P^−^) and 05 individuals with leprosy and with periodontitis (L^+^P^+^). Titrations with a pool containing 03 samples without leprosy and without periodontitis (L^−^P^−^) and another with 03 samples without leprosy and with periodontitis (L^−^P^+^) were also prepared, using the same conditions as those employed in the pools with leprosy.

The samples were tested in triplicate using two antigen concentrations, two saliva dilutions and antibody conjugated with peroxidase, as described in Table 1. The optic density coefficients (OD) were determined for each combination of antigen, saliva and conjugated antibody tested. Negative controls containing only the supernatant dilution buffer were also included.

**Table 1:** Initial antigen concentration, saliva dilution and conjugated dilution

Antigen	Antigen concentration	Saliva dilution	Conjugated dilution
*P. gingivalis* crude extract	2 µg/mL 5 µg/mL	1:2 1:50	1:1.000 1:5.000
HmuY	2 µg/mL 5 µg/mL	1:2 1:50	1:5.000 1:10.000

High binding polystyrene microplates (Greiner Bio-One, Frickenhausen, Alemanha) were sensitized with 50 µL of each diluted antigen in a 0.05 M carbonate-bicarbonate buffer, pH 9.6, per well, which were then incubated in a wet box at 8 °C for 15 h. The plates were washed twice with phospate buffering solution (PBS) containing 0.05% tween-20 detergent (PBS-T). Following this, the plates were blocked with 5% PBS skim milk, 200 L/well (Molico, Araçatuba, Brasil), and incubated for 2 h at 37 °C. They were then washed twice in PBS-T and the saliva supernatant diluted in 2% PBS skim milk (MOLICO®, Araçatuba, Brasil) added into wells and incubated at 37 °C for one hour. After five washings, the peroxidase conjugated anti-IgA was added and the plates incubated again for one hour at 37 °C (Trindade et al. 2012).

Next, after 5 washings with PBS-T, revelation was done using chromogen tetramethylbenzidine (TMB) (50 µL/well) in each pool and incubated for 10 min at room temperature and avoiding direct light. The reactions were interrupted by adding H_2_SO_4_ 4N, 25 µL/well, before proceeding to optical density readings at 450 to 650 nm (Trindade et al. 2012) using an ELISA reader (Multiskan GO, Termo Fisher Scientific OY, Vantaa, Finland).

### Data analysis

For checkerboard titration analysis, the OD averages of the triplicates obtained from each pool were calculated. The ratios between the averages of the pools with and without periodontitis (L^+^P^+^/L^+^P^−^) in individuals with and without leprosy were obtained, generating the coefficient for each condition tested. The best condition was selected according to the highest coefficient obtained. This same condition was applied to obtain the coefficients of pools L^−^P^+^/ L^−^P^+^ and for validation test analysis.

The averages of the OD triplicates obtained from each sample were calculated for the construction of the ROC curve, which was used to obtain values for the area under the curve, significance levels and confidence intervals. The cut-off point and sensitivity and specificity values for the validation of each test were assessed. The data was analyzed using SPSS version 23, with p ≤ 0.05 considered as statistically significant. The graphics were generated in EXCEL for Windows® program.

## Results

The standardization of ELISA was executed with supernatant pools containing the saliva of 10 individuals diagnosed with leprosy, with and without periodontitis (L^+^P^+^ and L^+^P^−^), with an average age of 46, 70 years, with a standard deviation of 14.94, being 05 men and 05 women. Saliva samples from 56 individuals with leprosy (24P^+^ and 19P^−^), 43.78 ± 13.17, 32 men and 24 women were used for the validation test.

**Fig. 1:**
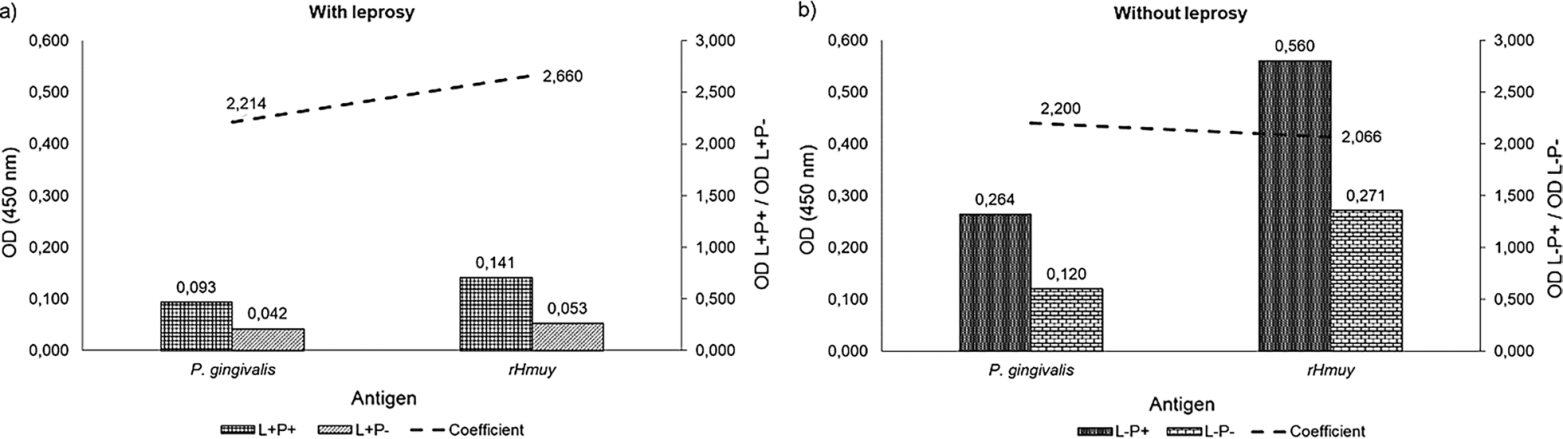
**a** Average optic density for pools of individuals with leprosy and with periodontitis (L^+^P^+^) and individuals with leprosy and without periodontitis (L^+^P^−^). **b** Average optic density for pools of individuals without leprosy and with periodontitis (L^−^P^+^) and individuals without leprosy and without periodontitis (L^−^P^−^)

The coefficients obtained demonstrated that the standardized tests make it possible to distinguish between the pools, both for individuals L^+^P^+^ and L^+^P^−^ and the individuals in L^−^P^+^ and LP^−^ (Fig. 1). In the *P. gingivalis* crude extract ELISA, the greatest coefficients, for individuals with and without leprosy, were obtained using the antigen at a concentration of 2 µg/mL, saliva diluted in 1:50 and conjugated antibody diluted in 1:5000. In the tests with HmuY, the best conditions were with antigen 2 µg/mL, saliva 1:2 and conjugated antibody in 1:10,000.

Among the individuals with leprosy, the coefficient among individuals with and without periodontitis was 2.21 for the test carried out with *P. gingivalis crude extract* and 2.66 for the test with HmuY. In the tests conducted with individuals without leprosy, the coefficients were 2.2 and 2.06 for the *P. gingivalis* crude extract and HmuY, respectively.

**Fig. 2:**
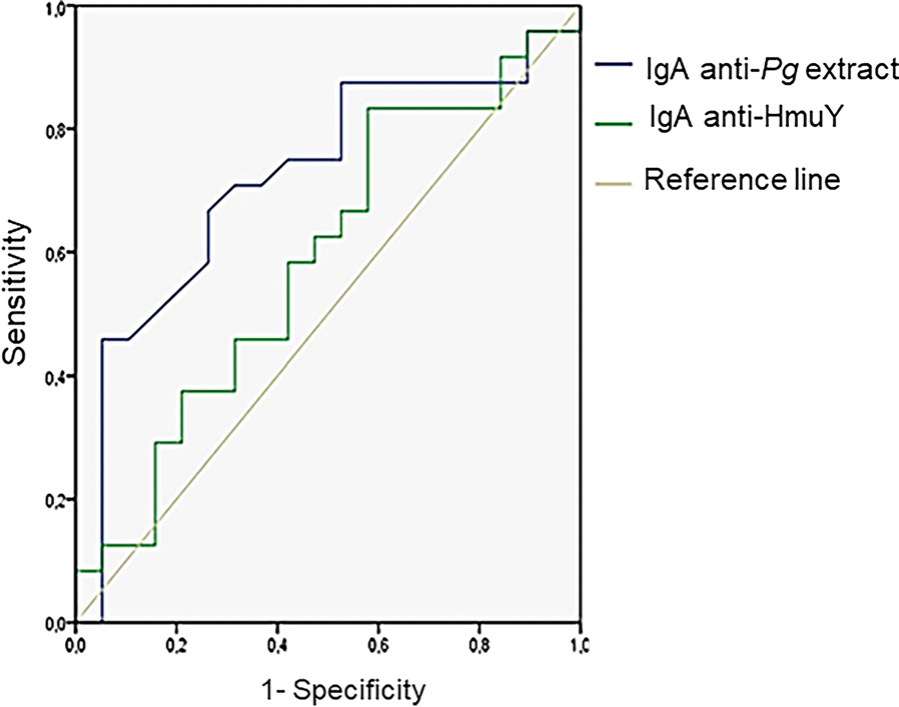
ROC curve constructed with the OD triplicate averages obtained for each sample. In blue, the curve for the *P. gingivalis* crude extract antigen: area under the curve = 0.720; p = 0.014; CI 0.56–0.88, cut-off 0.134, sensitivity = 66.7% and specificity = 73.7%. In green, the curve for HmuY antigen: area under the curve = 0.588; p = 0.328; CI 0.41–0.76, cut-off = 0.134, sensitivity+ 62.5% and specificity = 52.6%

Given the optimal conditions achieved, the validation tests conducted on samples from individuals with leprosy demonstrate that *P. gingivalis* crude extract antigen presented the best quality parameters in the ROC curve: area under the curve = 0.720; p = 0.014; CI 0.56–0.88. With these parameters, the sensitivity and specificity values were 66.7% and 73.7%, respectively, with a cut-off of 0.134 (Fig. 2).

The parameters obtained with the ROC curve for the HmuY ELISA do not demonstrate good discrimination between individuals with and without periodontitis: area under the curve = 0.588; p+ 0.328; CI 0.41–0.76, with 62.5% sensitivity, 52.6% specificity and a 0.134 cut-off.

## Discussion

Both *P. gingivalis* crude extract and HmuY protein were recognized by specific salivary IgA in individuals with and without leprosy. However, the specific recognition of *P. gingivalis* crude extract was stronger in the samples of individuals with periodontitis than in those without this oral disease, which makes this validated test an apt tool for the study of periodontitis in individuals with leprosy. In contrast, the HmuY recombinant protein did not show an equal performance.

Although both antigens were recognized, the first, made up of somatic proteins of the bacteria, seems to favor a more specific recognition, possibly due to the salivary IgA, which had undergone affinity maturation, found in individuals who had more previous contact with the bacteria, that is, those clinically diagnosed with periodontitis (Carvalho-Filho et al. 2019). Regarding HmuY, as it is an isolated protein, it is possible that individuals with periodontitis were not sensitized to this protein at the moment of pathogen-host interaction (Śmiga et al. 2015). It should be stressed that it is not a constitutive protein of the bacterium. Rather, it is expressed when there is lack of iron in the environment (Hägewald et al. 2002).

ELISA checkerboard tritation made it possible to determine where the antigen and antibody concentrations meet to achieve an equivalence zone, in the responses of healthy and diseased individuals. This method favors the possibility of eliciting responses with high titers in individuals with greater previous exposure to the antigen, while those with less previous exposure responded with low levels of antibodies.

It is worth noting that the use of ELISA for investigations concerning the biological plausibility of an association between two conditions, such as periodontitis and leprosy reactions in individuals diagnosed with leprosy, is of interest, as it is easily executed, low cost and has good reproducibility (Lin 2015). Furthermore, saliva collection is simple, avoiding invasive procedures. It is known that saliva marker analyses reflect a local response, result of a bacteria–host interaction in periodontal tissues (Matos et al. 2018; Carvalho-Filho et al. 2019).

On the other hand, saliva can contain contaminants, such as food and cosmetic micro-residues, which interfere in test sensitivity, as well as presenting some limitations, as it is a fluid with low stability due to the presence of enzymes (Matos et al. 2018). In the present study we tried to minimize the contamination with micro-residues collecting the saliva one hour after meal. The use of protease inhibitor was an strategy employed to maintain the stability of the sample. Additionally, antigens taken from bacterial extract can suffer compositional variations depending on the cultivation condition employed, which can reflect indirectly in the ELISA result. The use of recombinant proteins could overcome the problem with theses variations.

It is important to note that the test validation was carried out in individuals with leprosy, which may have interfered in the performance of the test, especially in relation to sensitivity. Studies have shown that IgG and IgA levels were higher in patients with chronic periodontitis than in healthy individuals. The average levels of serum IgG and salivary IgA increased as the seriousness of the disease increased (Gadekar et al. 2018; Carvalho-Filho et al. 2019).

The individuals in this study sample were undergoing PQT treatment or taking drugs to control leprosy reactions, which may have interfered in the bacterial load, consequently, modulating the humoral response. These drugs can also reduce saliva flow, influence the composition of saliva or provoke systemic and local alterations that impact on the buccal cavity (Femiano et al. 2008), and, as such, the findings should be treated with caution.

However, it is worth noting that before this study there was no previous evidence that had analyzed salivary IgA antigen specific to *P. gingivalis* levels in individuals with leprosy, with the exception of some studies concerning diabetes, acute alcoholic hepatitis and other systemic conditions (Mysak et al. 2014). Therefore, a comparison group was used to evaluate the IgA formed by individuals without leprosy diagnosis, as gold standard, with and without periodontitis, so as to ascertain the best conditions for the test employed, free from the response interference provoked by this chronic infectious neurological disease.

Based on the above considerations, indirect ELISA can be used as a tool to detect humoral immune response against *P. gingivalis* and its virulence factors, contributing, as such, to an avaluation of the association between periodontitis and leprosy.

This standardized ELISA to detect levels of salivary IgA antibody specific to *P. gingivalis* antigens was capable of discriminating between individuals with periodontitis and without periodontitis and with a leprosy diagnosis.

## Data Availability

The data of this work are available at: http://www.labimuno.ufba.br/.

## Code availability

Not applicable for that section.

## References

[CR1] Almeida JRS, Alencar CH, Barbosa JC, Dias AA, Almeida MEL (2013). Autopercepção de pessoas acometidas pela hanseníase sobre sua saúde bucal e necessidade de tratamento. Cien Saude Colet.

[CR2] Bachrach G, Muster Z, Raz I, Chaushu G, Stabholz A, Nussbaum G, Gutner M, Chaushu S (2008). Assessing the levels of immunoglobulins in the saliva of diabetic individuals with periodontitis using checkerboard immunodetection. Oral Dis.

[CR3] Bachtiar EW, Putri AC, Bachtiar BM (2020). Salivary nitric oxide, simplified oral hygiene index, and salivary flow rate in smokers and non-smokers: a cross-sectional study. F1000Res.

[CR4] Brasil (2016) Diretrizes para vigilância, atenção e eliminação da Hanseníase como problema de saúde pública: manual técnico-operacional. Ministério da Saúde, Brasília. https://portalarquivos2.saude.gov.br/images/pdf/2016/fevereiro/04/diretrizes-eliminacao-hanseniase-4fev16-web.pdf. Accessed 05 Feb 2021

[CR5] Cardoso EM, Reis C, Manzanares-Céspedes MC (2018). Chronic periodontitis, inflammatory cytokines, and interrelationship with other chronic diseases. Postgrad Med.

[CR6] Carvalho-Filho PC, Moura-Costa LF, Pimentel ACM, Lopes MPP, Freitas SA, Miranda PM, Costa RS, Figueirêdo CAV, Meyer R, Gomes-Filho IS, Olczak T, Xavier MT, Trindade SC (2019). Apoptosis transcriptional profile induced by *Porphyromonas gingivalis* HmuY. Mediators Inflamm.

[CR7] Chapple ILC, Bouchard P, Cagetti MG, Campus G, Carra MC, Cocco F, Nibali L, Hujoel P, Laine ML, Lingström P, Manton DJ, Montero E, Pitts N, Rangé H, Schlueter N, Teughels W, Twetman S, Van Loveren C, Van der Weijden F, Vieira AR, Schulte AG (2017). Interaction of lifestyle, behavior or systemic diseases with dental caries and periodontal diseases: consensus report of group 2 of the joint EFP/ORCA workshop on the boundaries between caries and periodontal diseases. J Clin Periodontol.

[CR8] Femiano F, Lanza A, Buonaiuto C, Gombos F, Rullo R, Festa V, Cirillo N (2008). Oral manifestations of adverse drug reactions: guidelines. J Eur Acad Dermatol Venereol.

[CR9] Franca M, Moura-Costa L, Meyer RJ, Trindade SC, Tunes UR, Freire SM (2007). Humoral immune response to antigens of *Porphyromonas gingivalis* ATCC 33277 in chronic periodontitis. J Appl Oral Sci.

[CR10] Gadekar NB, Hosmani JV, Bhat KG, Kotrashetti VS, Nayak RS, Babji DV, Pattanshetty SM, Joshi VM, Bansode RA (2018). Detection of antibodies against *Aggregatibacter actinomycetemcomitans* in serum and saliva through ELISA in periodontally healthy individuals and individuals with chronic periodontitis. Microb Pathog.

[CR11] Garlet GP (2010). Destructive and protective roles of cytokines in periodontitis: a re-appraisal from host defense and tissue destruction viewpoints. J Dent Res.

[CR12] Gaschignard J, Grant AV, Thuc NV, Orlova M, Cobat A, Huong NT, Ba NN, Thai VH, Abel L, Schurr E, Alcaïs A (2016). Pauci- and multibacillary leprosy: two distinct, genetically neglected diseases. PLoS Negl Trop Dis.

[CR13] Gomes-Filho IS, Trindade SC, Passos-Soares JS, Figueiredo ACMG, Vianna MIP, Hintz AM, Batista JET, Orrico GS, Conceição SS, Coelho JMF, Santos PNP, Nascimento MT, Miranda SS, Ramos MX, Porto ECL, Cruz CA, Carvalho SS, Cruz SS (2018). Clinical diagnosis criteria for periodontal disease: an update. J Dent Health Oral Disord Ther.

[CR14] Hägewald S, Bernimoulin J-P, Köttgen E, Kage A (2002). Salivary IgA subclasses and bacteria-reactive IgA in patients with aggressive periodontitis. J Periodontal Res.

[CR15] Krasse B (1988). Risco de cáries: guia prático para controle e assessoramento.

[CR16] Lin AV (2015). Indirect ELISA. Methods Mol Biol.

[CR17] Matos FZ, Aranha AMF, Borges ÁH, Pedro FLM, Raslan SA, Hamida F, Veiga K, Porto AN (2018). Can different stages of leprosy treatment influence the profile of oral health? Oral status in leprosy. Med Oral Patol Oral Cir Bucal.

[CR18] Mira A, Simon-Soro A, Curtis MA (2017). Role of microbial communities in the pathogenesis of periodontitis and caries. J Clin Periodontol.

[CR19] Motta ACF, Furini RB, Simão JCL, Vieira MB, Ferreira MAN, Komesu MC, Foss NT (2011). Could leprosy reaction episodes be exacerbated by oral infections?. Rev Soc Bras Med Trop.

[CR20] Mysak J, Podzimek S, Sommerova P, Lyuya-Mi Y, Bartova J, Janatova T, Prochazkova J, Duskova J (2014). *Porphyromonas gingivalis*: major periodontopathic pathogen overview. J Immunol Res.

[CR21] Naaz F, Mohanty PS, Bansal AK, Kumar D, Gupta UD (2017). Challenges beyond elimination in leprosy. Int J Mycobacteriol.

[CR22] Odriozola EP, Quintana AM, González V, Pasetto RA, Utgés ME, Bruzzone OA, Arnaiz MR (2017). Towards leprosy elimination by 2020: forecasts of epidemiological indicators of leprosy in Corrientes, a province of northeastern Argentina that is a pioneer in leprosy elimination. Mem Inst Oswaldo Cruz.

[CR23] Olczak T, Siudeja K, Olczak M (2006). Purification and initial characterization of a novel *Porphyromonas gingivalis* HmuY protein expressed in *Escherichia coli* and insect cells. Protein Expr Purif.

[CR24] Olczak T, Sosicka P, Olczak M (2015). HmuY is an important virulence factor for *Porphyromonas gingivalis* growth in the heme-limited host environment and infection of macrophages. Biochem Biophys Res Commun.

[CR25] Papapanou PN, Sanz M, Buduneli N, Dietrich T, Feres M, Fine DH, Flemmig TF, Garcia R, Giannobile WV, Graziani F, Greenwell H, Herrera D, Kao RT, Kebschull M, Kinane DF, Kirkwood KL, Kocher T, Kornman KS, Kumar PS, Loos BG, Machtei E, Meng H, Mombelli A, Needleman I, Offenbacher S, Seymour GJ, Teles R, Tonetti MS (2018). Periodontitis: consensus report of workgroup 2 of the 2017 world workshop on the classification of periodontal and peri-implant diseases and conditions. J Periodontol.

[CR26] Pudakalkatti PS, Baheti AS (2015). Correlation of salivary immunoglobulin A against lipopolysaccharide of *Porphyromonas gingivalis* with clinical periodontal parameters. Contemp Clin Dent.

[CR27] Schreuder PAM, Noto S, Richardus JH (2016). Epidemiologic trends of leprosy for the 21st century. Clin Dermatol.

[CR28] Śmiga M, Bielecki M, Olczak M, Smalley JW, Olczak T (2015). Anti-HmuY antibodies specifically recognize *Porphyromonas gingivalis* HmuY protein but not homologous proteins in other periodontopathogens. PLoS ONE.

[CR29] Teixeira MAG, Silveira VM, França ER (2010). Características epidemiológicas e clínicas das reações hansênicas em indivíduos paucibacilares e multibacilares, atendidos em dois centros de referência para hanseníase, na Cidade de Recife, Estado de Pernambuco. Rev Soc Bras Med Trop.

[CR30] Trindade SC, Olczak T, Gomes-Filho IS, Moura-Costa LF, Cerqueira EMM, Galdino-Neto M, Alves H, Carvalho-Filho PC, Xavier MT, Meyer R (2012). Induction of interleukin (IL)-1b, IL-10, IL-8 and immunoglobulin G by *Porphyromonas gingivalis* HmuY in humans. J Periodontal Res.

[CR31] Walker SL, Lockwood DNJ (2006). The clinical and immunological features of leprosy. Br Med Bull.

[CR32] World Health Organization (2017) Guidelines for the diagnosis, treatment and prevention of leprosy. World Health Organization, Regional Office for South-East Asia, New Delhi. https://apps.who.int/iris/handle/10665/274127. Accessed 12 Feb 2021

